# A simple and evolutional approach proven to recanalise the nasolacrimal duct obstruction

**DOI:** 10.1136/bjo.2008.149393

**Published:** 2009-05-04

**Authors:** D Chen, J Ge, L Wang, Q Gao, P Ma, N Li, D-Q Li, Z Wang

**Affiliations:** 1 Department of ENT, First Affiliated Hospital, Sun Yat-sen University, Guangzhou, PR China; 2 State Key Laboratory of Ophthalmology, Zhongshan Ophthalmic Center, Sun Yat-sen University, Guangzhou, PR China; 3 Cullen Eye Institute, Department of Ophthalmology, Baylor College of Medicine, Houston, Texas, USA

## Abstract

**Aim:**

To evaluate a new approach of recanalisation of nasolacrimal duct obstruction (RC-NLDO) in the treatment of the nasolacrimal duct obstruction (NLDO) and chronic dacryocystitis.

**Methods:**

583 patients with 641 eyes suffering from NLDO and chronic dacryocystitis were enrolled in this study. The RC-NLDO was performed in 506 eyes, with 135 eyes undergoing external dacryocystorhinostomy (EX-DCR) as controls. Patient follow-up for 54 months was evaluated by symptoms, dye disappearance test, lacrimal irrigation and digital subtraction dacryocystogram. The RC-NLDO was also performed in 12 rhesus monkeys for histopathological examination.

**Results:**

The clinical success rates were 93.1% in 506 cases of RC-NLDO and 91.11% in 135 cases of EX-DCR. The success rates for second surgery were achieved in 85.19% on RC-NLDO and 40.0% on EX-DCR. No major intra- or postoperative complications were observed in the RC-NLDO group. The mean operative duration was 12.5 min for RC-NLDO and 40.3 min for EX-DCR (p<0.001). A pathological study in rhesus monkeys demonstrated that the RC-NLDO wounded epithelium in nasolacrimal duct healed completely within 1 month without granulation tissue formation.

**Conclusion:**

The findings demonstrate that the RC-NLDO is a simple and effective approach proven to recanalise the obstructed nasolacrimal duct with a comparable success rate to EX-DCR.

Nasolacrimal duct obstruction (NLDO) and chronic dacryocystitis are common ophthalmic diseases. The external dacryocystorhinostomy (EX-DCR) has been the most effective and standard surgery in treating these conditions since 1904 when it was reported by Toti.^1^ However, EX-DCR is an invasive, relatively complex and time-consuming procedure that causes a facial cutaneous scar. Many patients prefer to suffer tearing rather than undergo this surgery.^2^ ^3^ The improvement on DCR has been made recently, such as the endonasal DCR and endocanalicular laser DCR. These approaches were promising but still necessitate bone removal and require costly equipment. These surgical procedures were reported to have less effective results than EX-DCR and involve a marked learning curve.^4^ ^5^ ^6^ ^7^ ^8^ ^9^ The approach of the EX-DCR and these new procedures is to create a bypass draining system, rather than to restore the obstructed nasolacrimal duct.

Recanalisation of nasolacrimal duct obstruction (RC-NLDO) was an evolutionally developed surgical approach for treating these conditions to restore the native nasolacrimal duct, using a simple instrument, the lacrimal canaliser, which we created in 1994.^10^ Since then, this approach has been widely adopted by many ophthalmologists in China for its simplicity, safety, efficacy and minimal invasion.^11^ ^12^ ^13^ ^14^ ^15^ In the present study, we report the long-term follow-up results of RC-NLDO in the clinical treatment for 506 cases of NLDO and chronic dacryocystitis, as well as the histopathological evidence from animal experiments. The relative indication, contraindication, surgical technique, postoperative care, complications, advantages and disadvantages of the RC-NLDO are discussed.

## Materials and methods

### Patients

This study adhered to the tenets of the Declaration of Helsinki and was approved by the Institutional Review Board (IRB)/Ethics Committee of Zhongshan Ophthalmic Center, Sun Yat-sen University. All cases were chosen from outpatients who were diagnosed as having NLDO and/or chronic dacryocystitis. Every patient underwent preoperatively comprehensive ophthalmic and intranasal examination. Dacryocystogram or digital subtraction dacryocystogram was performed in some cases.

A total of 641 eyes of 583 consecutive patients were recruited from July 2003 to June 2006 with their signed informed consent forms, including 135 eyes of 126 patients undergoing the EX-DCR and 506 eyes of 457 patients undergoing the RC-NLDO. There were no statistical differences in patient demographics between these two groups. The male-to-female ratio was approximately 1:3, and the average age was 50 years. The duration of symptoms ranged from 6 months to 26 years (mean 5.1 years) in the RC-NLDO group and from 6 months to 17 years (mean 4.7 years) in the EX-DCR group.

### Instrument used for RC-NLDO

**Figure 1:**
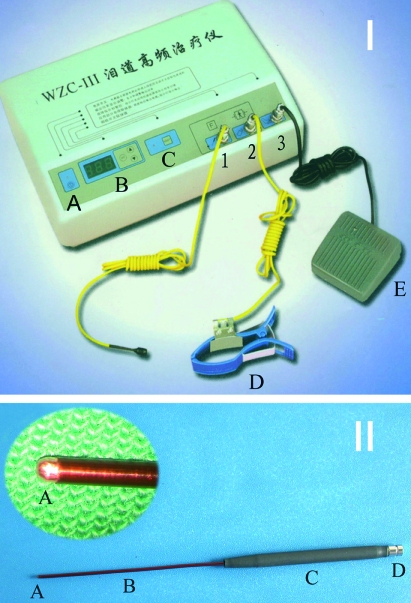
Lacrimal canaliser (model WZC-III) with accessories. (I) Main instrument containing an on/off switch (A), an output power control (B) and a reset button (C) with three cable connectors (1, 2 and 3) for connections to accessories: a positive electrode cable that links to the high-frequency lacrimal probe (see part II), negative electrode tongs (D) and foot pedal (E), respectively. (II) Lacrimal probe images showing: (A) its 2.0 mm long, naked (without an insulating coat on the surface) and conducting tip 1.2 mm in diameter; (B) an 80 mm long and 1.2 mm diameter probe body with a thin layer of non-toxic and insulating coat on the surface; (C) a 60 mm long and 5 mm diameter head part of the probe covered by a rubber layer; (D) a special “buckle” structure of the probe top.

The instrument used for recanalisation of nasolacrimal duct obstruction was the lacrimal canaliser consisting of a console and its accessories (fig 1). The console can discharge a power current (50–150 W) with 500 kHz frequency. The high-frequent lacrimal (HFL) probe is made of copper–silver alloy 1.2 mm in diameter and 140 mm in length. Its tip is 2.0 mm long, smooth, blunt and naked (without an insulating coat on the surface), features allowing it to cauterise blocked tissue in a nasolacrimal duct.

### Surgical procedures

EX-DCR was performed under local anaesthesia in a standardised fashion.^9^ The RC-NLDO was performed under local infiltration anaesthesia with 2% lidocaine hydrochloride. The inferior nasal meatus was treated twice with a pledget soaked in 0.5% Alcaine eye-drops (Alcon, Fort Worth, Texas) and 1% ephedrine hydrochloride solution, and then packed with the same pledget to protect the nasal bottom. The HFL probe was inserted into the nasolacrimal duct until the packed pledget was moving, a indication that the probe tip was into the nasal cavity. Then, the electrocauterisation was performed during the time when the probe pulled back in a slowly retrograde way. The blocked tissue in the nasolacrimal duct was easily cauterised by the energy and became a charred crust tube. The cauterisation was stopped when the probe was almost out of the nasolacrimal duct. The HFL probe was then reinserted into the nasolacrimal duct to check any remaining obstruction. If there was any resistance, the electrocauterisation procedure was repeated until the HFL probe passed freely and smoothly through the nasolacrimal duct. The lacrimal drainage system was then irrigated with antibiotic solution.

### Postoperative care

All patients were prescribed topical antibiotic eye-drops and a nasal mucosa astringent four times a day for 10 days. The lacrimal passage was irrigated with antibiotic solution weekly in the first half month after surgery. Postoperative evaluation and long-term follow-up were performed by the same doctors.

### Criteria defining the clinical effects

Clinical success was defined by the results of the dye disappearance test, lacrimal irrigation and symptoms. “Full success” was noted if the fluorescent stain was positive in 5 min, indicating the free nasolacrimal passage, and the symptoms were completely resolved. The lacrimal irrigation was performed if no fluorescent stain was found, or the stain was found after more than 10 min. “Partial success” was noted if the pledget was stained with fluorescein after lacrimal irrigation and there was no reflux. “Partial success” was also scored for the patient who had some symptoms but no reflux in lacrimal irrigation. “Failure” was defined as no improvement or recurrence in tearing with severe reflux in irrigation at the last follow-up.

### Histopathological study after RC-NLDO in rhesus monkeys

Twelve rhesus monkeys (1.5–2 years old and weighing 4–6 kg) were purchased from Guangdong Medical Laboratory Animal Center, Guangdong, China. Experimental procedures were performed adhering to the ARVO statement for the Use of Animals in Ophthalmic and Vision Research. One eye randomly chosen from each monkey was used in RC-NLDO, and the other eye from each animal was used as an untreated normal control. The surgery and postoperative care were performed in the same manner as for the patients described above. The animals were killed by an overdose of barbiturates 7 days, 1 month, 2 months and 3 months after surgery. Specimens including canaliculi, lacrimal sac and nasolacrimal duct were carefully collected for histopathological examination.

## Results

### Patient preoperative conditions

The patient preoperative conditions are summarised in table 1. The duration of symptoms ranged from 6 months to 26 years (mean 5.1 years) in the RC-NLDO group, and from 6 months to 17 years (mean 4.7 years) in the EX-DCR group. There were 49 (9.67%) recurrent cases suffering tearing from previously unsuccessful surgery (EX-DCR or silicone intubation) in the RC-NLDO group, and 10 (7.41%) recurrent cases in the EX-DCR group. The follow-up period after surgeries ranged from 12 to 54 months (mean 28.5 months).

**Table 1:** Preoperative conditions of patients in two groups

Preoperative conditions	Recanalisation of nasolacrimal duct obstruction	External dacryocystorhinostomy
	n (percentage of total (506))	n (percentage of total (135))
Total	506 (100.0%)	135 (100.0%)
Nasolacrimal duct obstruction	125 (24.80%)	51 (37.78%)
Chronic dacryocystitis	255 (50.40%)	72 (53.33%)
Mucocoele	14 (2.77%)	2 (1.48%)
Fistulae	10 (1.98%)	0 (0%)
Small lacrimal sac	45 (8.80%)	0 (0%)
Atrophic rhinitis	8 (1.58%)	0 (0%)
Failed in external dacryocystorhinostomy	23 (4.55%)	3 (2.22%)
Failed in silicone intubation	26 (5.14%)	7 (5.19%)

### Clinical effects of the RC-NLDO and EX-DCR treatments

**Figure 2:**
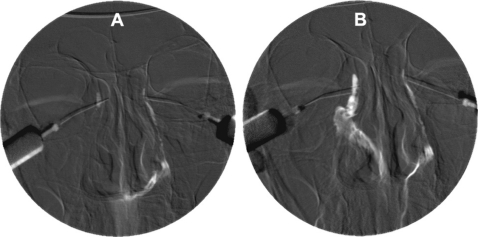
Digital subtraction dacryocystogram. (A) Completely obstructed right nasolacrimal duct and a normal left one before recanalisation of nasolacrimal duct obstruction (RC-NLDO) surgery. (B) Free flow of the contrast medium through the recanalised nasolacrimal duct to the inferior meatus 3 weeks after RC-NLDO operation.

The surgical outcomes are summarised in table 2. The operative duration for RC-NLDO ranged from 8 to 19 min (12.5 (SD 2.6) min), significantly shorter than the 30–50 min (40.3 (4.7) min) for the EX-DCR group (p<0.001, Student t test). In the RC-NLDO group, the full success was defined in 440 (86.96%) eyes, partial success in 31 (6.13%) cases and recurrent in 35 (6.92%) cases. The total success rate reached 93.08% (471/506) with a single treatment. A total of 27 failed cases underwent a second repeated surgery 3 months later. After second surgery, 20 (74.07%) cases achieved full success; three (11.11%) cases achieved partial success, and only four (14.81%) cases failed again. The total success rate of second repeated surgery was 85.19%. In the EX-DCR group, complete success with symptoms completely resolved was achieved in 118 (87.14%) cases, partial success in five (3.70%) cases and failure in 12 (8.89%) cases. The total success rate reached 91.11% (123/135). A total of 10 failed cases underwent repeated surgery, in which four (40.0%) cases were successful, and six (60.0%) cases had failed. There was no statistical difference in surgical outcomes between these two groups for the primary surgery (p = 0.816), while a significant difference was found for the recurrent patients (p<0.013). The digital subtraction dacryocystogram showed that the reconstructed cavity of the nasolacrimal duct by RC-NLDO was much wider than normal (fig 2).

**Table 2:** Clinical outcome in two groups

Variable	Recanalisation of nasolacrimal duct obstruction	External dacryocystorhinostomy	
	n (%)	n (%)	
Primary surgery	506 (100.0)	135 (100.0)	p*
Full success	440 (86.96)	118 (87.41)	
Partial success	31 (6.13)	5 (3.70)	
Total success	471 (93.08)	123 (91.11)	
Failure	35 (6.92)	12 (8.89)	
Second surgery	27 (100.0)	10 (100.0)	p†
Full success	20 (74.07)	4 (40.0)	
Partial success	3 (11.11)	0 (0)	
Total success	23 (85.19)	4 (40.0)	
Failure	4 (14.81)	6 (60.0)	

*p = 0.816, two-sample t test (a = 0.05).

**p = 0.013, two-sample t test (a = 0.05).

### Complications of RC-NLDO and EX-DCR

In the EX-DCR group, one patient (0.74%) suffered postoperative bleeding immediately after surgery; eight patients (5.93%) reported transient pain in the upper segment of the maxillary bone, but it was tolerable; and 30 patients (22.22%) complained about their visible scars. No infection or uncontrollable bleeding occurred. In the RC-NLDO group, four patients (0.79%) had postoperative periocular subcutaneous haematoma. No other complications were observed in this group.

### Histopathological results of rhesus monkeys undergoing RC-NLDO

**Figure 3:**
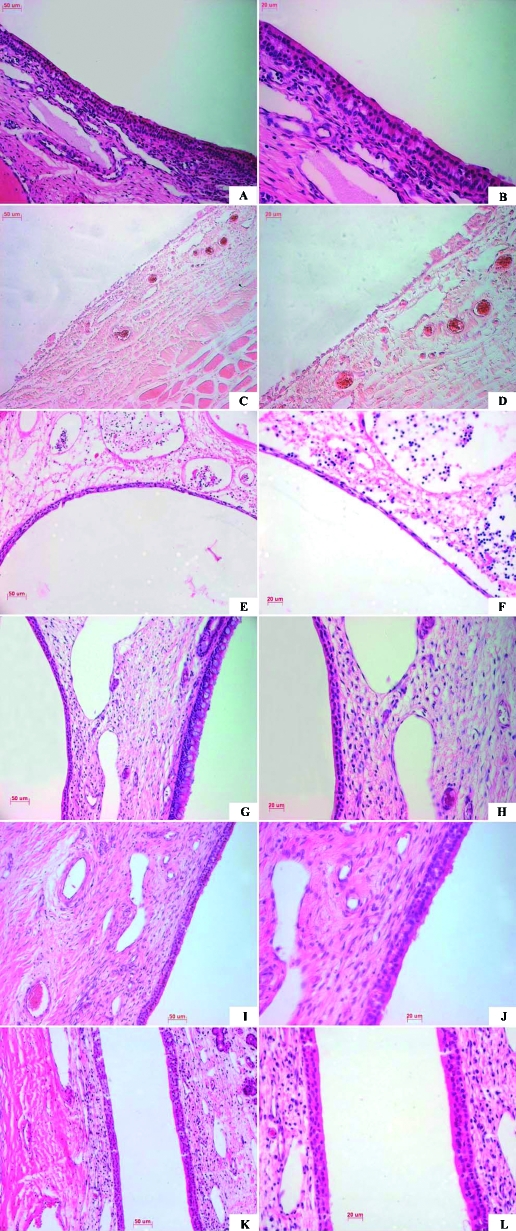
Representative images showing histomorphological structures of the nasolacrimal duct mucosa in cross-sections of rhesus monkeys before and after recanalisation of nasolacrimal duct obstruction (RC-NLDO) surgery. (A, B) Normal morphological structure of nasolacrimal duct mucosa in rhesus monkey; (C, D) nasolacrimal duct mucosa in rhesus monkey immediately after RC-NLDO surgery, showing almost total loss of epithelium in nasolacrimal duct with a few residual cells on the basal membrane. (E, F) Notable migration of cells from adjacent residual epithelia, 1 week after surgery. The epithelial cells formed a single layer and loosely covered the surface of the basement membrane. Scattered or focal infiltrations of inflammatory cells were visible in the lamina propria. (G, H) Completely healed epithelium with two layers of cells similar to the normal controls, 1 month after surgery. There was no visible inflammatory cell infiltration in lamina propria. (I, J) Two months after surgery. (K, L) Three months after surgery. The epithelia in specimens from 2 to 3 months later became morphologically and histologically normal. Magnification: ×200 in images A, C, E, G, I and K; ×400 in B, D, F, H, J and L.

In normal rhesus monkey, the lacrimal sac is lined by stratified columnar epithelium containing scattered goblet cells on a broad basement membrane while the nasolacrimal duct is lined by a double layer of epithelium, a superficial layer of columnar cells and a basal layer of non-keratinised squamous cells (fig 3A,B). It differs histologically from the lacrimal sac in that it lacks goblet cells.

The healing process of the epithelium in the nasolacrimal duct after RC-NLDO surgery in the rhesus monkey was evaluated by histopathological examinations. Cross-sections of the specimens obtained immediately after RC-NLDO displayed the intact epithelium of the lacrimal sac and an almost total loss of epithelium in the nasolacrimal duct with a few residual cells appearing as small islands on the basal membrane (fig 3C,D). Sections of the specimens collected 1 week after surgery showed notable migration of cells from adjacent residual epithelia. The epithelial cells formed a single layer and loosely covered the surface of the basement membrane. Scattered or focal infiltrations of inflammatory cells were visible in the lamina propria (fig 3E,F). Specimens from 1 month after surgery displayed a completely healed epithelium with two layers of cells similar to the normal controls. There was no visible inflammatory cell infiltration in the lamina propria (fig 3G,H). The epithelia in specimens from 2 to 3 months later became morphologically and histologically normal (fig 3I–L). No granulation tissue was noted in all specimens.

## Discussion

The ideal treatment for NLDO is to recanalise the obstructed duct and restore normal anatomical structure and physiological function of the lacrimal drainage system. The EX-DCR is a successful operation with a success rate of 80–95%, but it is a relatively complex procedure^6^ and involves skin incision and bone removal to create a mucosal fistula from the lacrimal sac directly into the nasal cavity, which leaves a facial cutaneous scar and disruption of the medial canthal anatomy.^1^ ^5^ ^6^ ^16^ ^17^

In order to overcome these disadvantages, a number of therapeutic developments and promising advances in DCR have been reported recently, such as endonasal (endoscopic) DCR and endocanalicular laser DCR.^9^ ^18^ However, these new techniques have obvious disadvantages, such as their time-consuming nature and a marked learning curve.^5^ ^6^ ^7^ ^8^ ^9^ In addition, the EX-DCR and these new approaches do not restore the obstructed nasolacrimal duct but make a bypass draining system, which is not a physiological tear passage.

Recanalisation of the nasolacrimal duct obstruction (RC-NLDO) was a simple and evolutional approach for treating NLDO and chronic dacryocystitis. In the last 5 years, we have been conducting this study with up to 54-month long-term follow-up to evaluate the RC-NLDO approach for comparison with EX-DCR. Our findings demonstrated that the RC-NLDO was a highly successful approach with an overall success rate at 93.18% for primary surgery and 85.19% for second repeated surgery. The pathological study in rhesus monkeys further confirmed that the surgically wounded epithelium in the nasolacrimal duct was starting to heal in a week and completely recovered within 1 month, creating a wide recanalised cavity.

RC-NLDO has achieved a high success rate that was comparable with EX-DCR. This may be due to the following factors. First a larger lumen was created (fig 2B). In laser treatment, the cavity created is narrower due to the limited diameter (0.4–0.6 mm) of the laser fibre. However, in RC-NLDO, the diameter of the HFL probe is 1.2 mm. According to the formula “S = πr^2^,” the reopened area (S = 1.13 mm^2^) of the duct cavity in cross-section by RC-NLDO was 4–9-fold larger than the laser-created cavity (0.13–0.28 mm^2^). According to the Poiseuille law, the flow resistance is the fourth power inversely proportional to the radius of the duct, the tear flow resistance through the cavity created by RC-NLDO would be 16–81-fold lower than that created by laser treatment. Second is the lower incidence of false passage formation. No false passage formation is essential for success. In normal conditions, the soft tissue of the membranaceous nasolacrimal duct adheres tightly to its surrounding osseous nasolacrimal duct. Therefore, when there is no false passage formation, the direction of surgical scar contraction is acentric, which pulls the soft tissue to the wall of the osseous nasolacrimal duct, so the gently acentric contraction of the surgical scar would not obstruct the cavity and would not reduce the success rate. If the false passage was formed, the direction of the surgical scar contract would pull itself towards the centre of the reconstructed nasolacrimal duct, which would narrow or block the cavity. The false passage could be avoided in most cases if the electrocauterisation was performed simultaneously while slowly withdrawing the HFL probe after its tip was inserted into the nasal cavity during the RC-NLDO procedure.

The RC-NLDO technique has several other advantages, such as (1) minimal trauma and no facial cutaneous scar due to the surgery being performed without cutaneous incision and bone excision; (2) less disruption of the lacrimal pump function due to the surgery restoring a physiological tear passage without making a bypass draining system; (3) a simpler, easier and faster (average 12.5 min) procedure similar to conventional lacrimal probing.

Usually, the lower canaliculus is ascendant in tear drainage (about 75%). Thus, the superior canaliculus was chosen for the HFL probe to pass through, which protected the function of tear drainage in case of any unexpected damage. The most obstructed points could be penetrated by rotational manipulation along with a slight force during electrocauterisation. In the procedure, the obstructed tissue was cauterised to an eschar crust tube tightly adhering to the wall of the reopened cavity, and became a transitorily sustaining membrane to the wall of the reconstructed nasolacrimal duct. Additionally, while the HFL probe is withdrawn, the rotational manipulation could keep the eschar crust tube intact and minimise the risk of postoperative bleeding, inflammation and synechia.

The RC-NLDO is also a choice of treatment for patients who suffer from a lacrimal sac mucocoele, obstructed lacrimal duct with atrophic rhinitis or small lacrimal sac. The RC-NLDO is suitable for patients who failed to respond to a previous EX-DCR.

RC-NLDO is not suitable for treating an obstructed osseous nasolacrimal duct which could be treated with EX-DCR. Contraindications for RC-NLDO also include acute dacryocystitis, suspicion of malignancy and patients suffering from severe hypertension or severe cardiac disease (especially with a pacemaker).

In conclusion, a simple and evolutional approach RC-NLDO has been evaluated by long-term follow-up in a large patient population and animal pathological examination, and the findings demonstrated that this new approach has been proven to recanalise the nasolacrimal duct obstructions. When compared with EX-DCR, RC-NLDO is a new option for treating NLDO and chronic dacryocystitis with a similar or better clinical success rate. The advantages include its efficacy, minimal invasion, safety and simplicity. RC-NLDO is also an optimal choice for recurrent patients who failed to respond to EX-DCR and for patients with small lacrimal sacs or atrophic rhinitis who are not suitable for EX-DCR.
